# Supplementary material for: Characterization of Anopheles gambiae immune cells through genetic and functional immunophenotyping
Source: Nat Commun. 2025 Dec 3;16:10875. doi: 10.1038/s41467-025-65895-6 (PMC12675505; doi:10.1038/s41467-025-65895-6)
Supplement: Supplementary file 1 — Supplementary Information [file 41467_2025_65895_MOESM1_ESM.pdf]

Supporting Information

Characterization of *Anopheles gambiae* immune cells through genetic and functional immunophenotyping

George-Rafael Samantsidis, Hyeogsun Kwon, and Ryan C. Smith

Department of Plant Pathology, Entomology and Microbiology, Iowa State University, Ames, IA, USA.

Included supporting information

Supplemental Figures

Supplementary Fig. 1. Overview of hemocyte promoter constructs.

Supplementary Fig. 2. Characterization of piggyBac insertions in transgenic *An. gambiae*.

Supplementary Fig. 3. Marker gene expression across different constructs and transgenic lines.

Supplementary Fig. 4. Additional characterization of the NimB2-CFP line.

Supplementary Fig. 5. Gene marker expression in response to clodronate liposome injection.

Supplementary Fig. 6. Co-localization of CFP and PPO6 in PPO6-CFP transgenic mosquitoes.

Supplementary Fig. 7. Visualization of LRIM15- and SCRASP1-GFP cells under native and fixed conditions.

Supplementary Fig. 8. Hemocyte-specific marker gene expression responses to blood feeding.

Supplementary Fig. 9. Blood-feeding promotes a shift in PPO6^{low} proportions.

Supplementary Fig. 10. Determination of gating for flow cytometry analysis.

Supplementary Fig. 11. Classification of immune cell subtypes in the P1-P5 DRAQ5 clusters.

Supplementary Fig. 12. Identification of fluorescent hemocyte populations by flow cytometry.

Supplementary Fig. 13. Determination of gating using fluorescent beads in flow cytometry.

Supplementary Fig. 14. Gating thresholds for analysis of phagocytosis in transgenic lines.

Supplemental Tables

Supplementary Table 1. Sequences of candidate hemocyte promoter constructs.

Supplementary Table 2. Comparison of gene expression and tissue specificity of transgenic lines.

Supplementary Table 3. Percentage of hemocytes expressing transgenic markers.

Supplementary Table 4. Primers used for the amplification of the hemocyte promoter regulatory regions.

Supplementary Table 5. Primers used in splinkerette PCR.

Supplementary Table 6. Primers used for gene expression analysis.

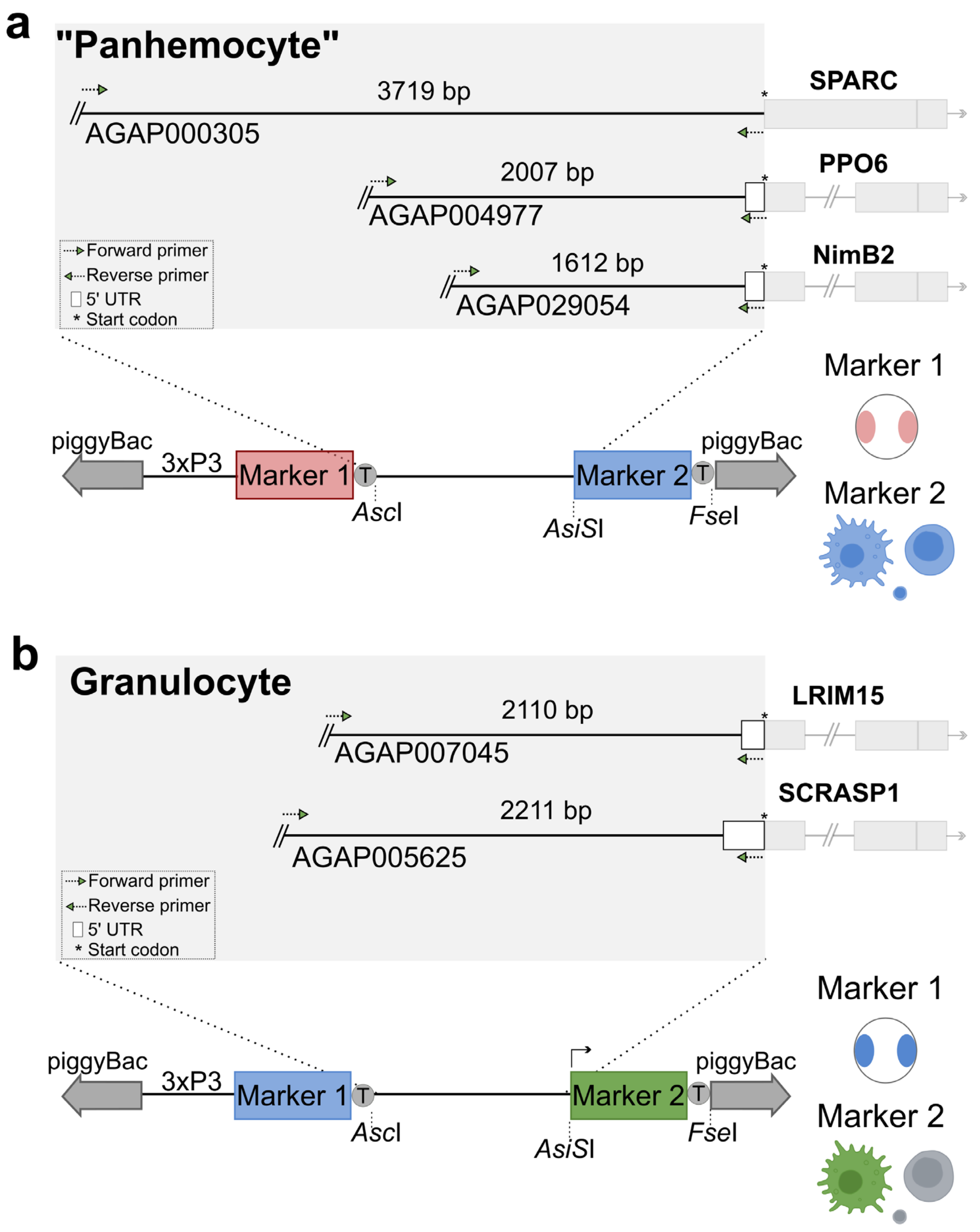

Supplementary Fig. 1. Overview of hemocyte promoter constructs. Schematic overview illustrating the constructs used to generate transgenic mosquitoes expressing fluorescent markers under the regulation of promoters with the intent to drive expression in all hemocyte populations (panhemocyte) (**a**) or specifically in granulocytes (**b**). For both **a** and **b**, the length of the putative promoter region (containing the 5' UTR) is displayed in base pairs (bp) and displays the respective genetic markers associated with each piggyBac construct. Graphical images in **a** and **b** were drawn by George-Rafael Samantsidis using Inkscape.

a

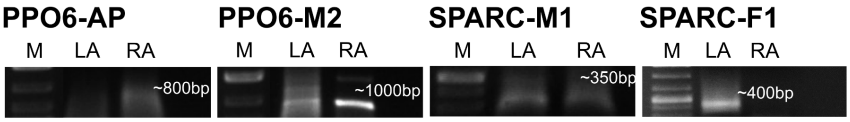

Strain	Line	Chr	Genomic coordinates	Associated Gene
**PPO6:CFP**	M2	2R	55710203-55710779	AGAP004403
	AP	X	9475427-9475283	AGAP000528
**SPARC:CFP**	M1	3R	18994307-18994439	AGAP008826
	F1	N/A	N/A	N/A
**NimB2:CFP**	MIB	N/A	N/A	N/A
	MIE	N/A	N/A	N/A

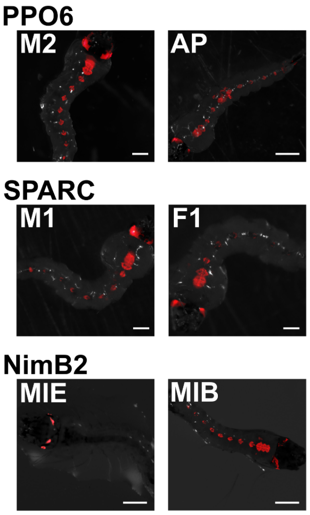

b

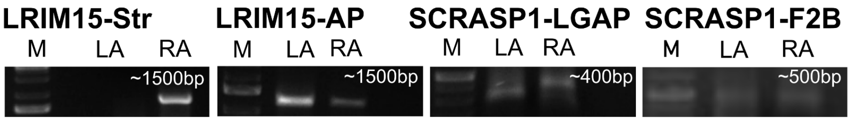

Strain	Line	Chr	Genomic coordinates	Associated Gene
**LRIM15:GFP**	Str	2R	5144656-5146010	AGAP001446
	AP	X	11617354-11614600	Intergenic
**SCRASP1:GFP**	LGAP	2R	29680727-29680903	AGAP002925
	F2B	2L	40518618-40518727	AGAP006994

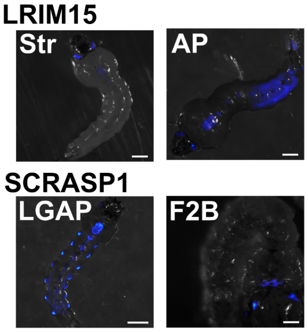

Supplementary Fig. 2. Characterization of piggyBac insertions in transgenic *An. gambiae*. The genomic insertions of each of the respective piggyBac hemocyte promoter constructs into the genome of *An. gambiae* were characterized using splinkerette PCR. For candidate panhemocyte (**a**) or granulocyte-specific (**b**) promoter constructs, gel images display PCR products for the left (LA)- or right (RA)-end PCR amplicons. Marker ladder, M. Genomic insertion sites and associated phenotypes produced from the insertion are displayed for the 3xP3 integration marker (RFP, **a**; CFP, **b**). Scale bars, 1mm. Uncropped gel images are provided as source data in a Source Data file.

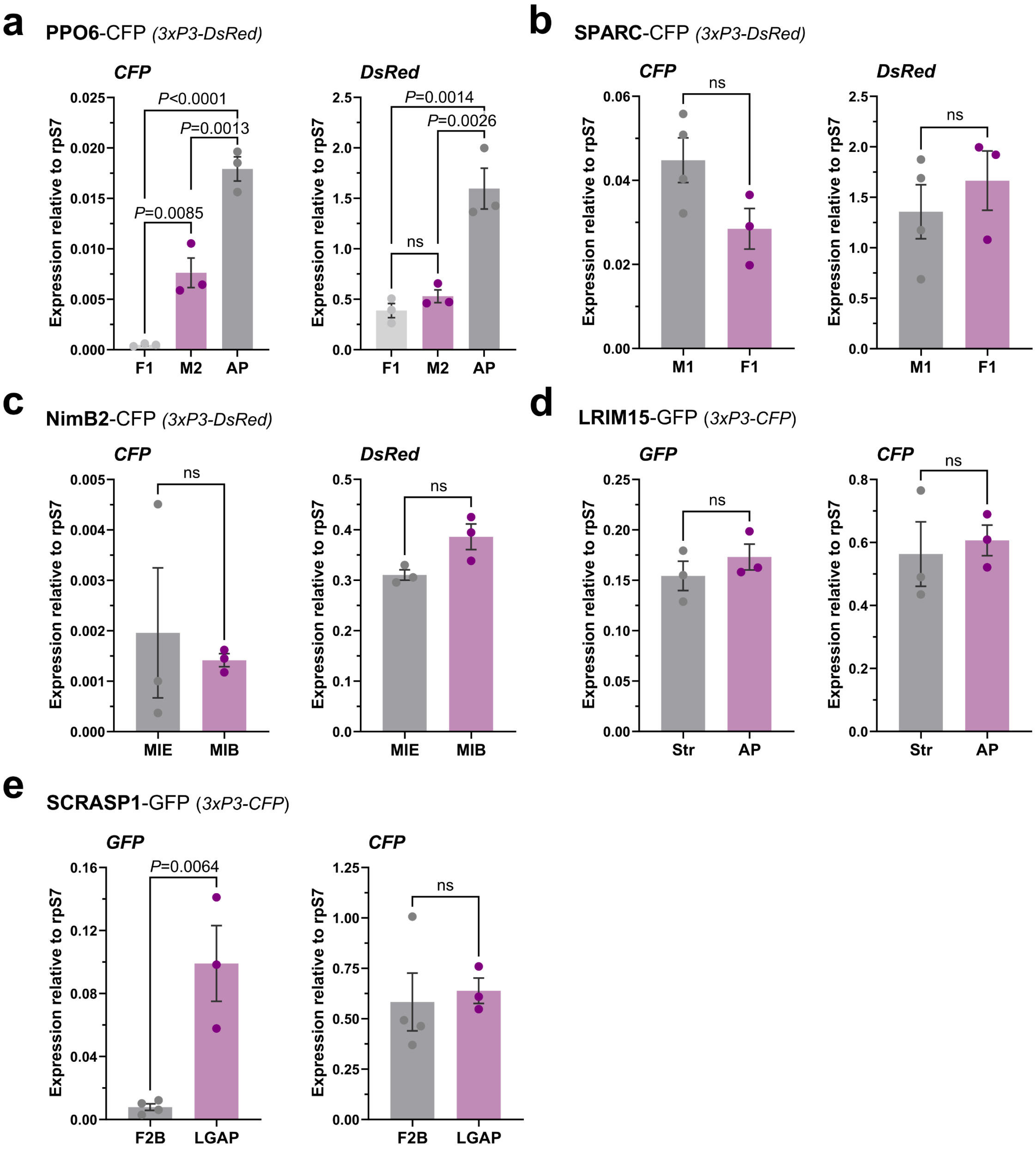

Supplementary Fig. 3. Marker gene expression across different constructs and transgenic lines. The expression profiles of each transgene and transgenic construct were examined in 3-5 days old naïve adult female mosquitoes (~10 pooled mosquitoes per replicate). Candidate panhemocyte promoter constructs, PPO6 (**a**), SPARC (**b**), and NimB2 (**c**), driving *CFP* expression under the control of the respective hemocyte promoter were evaluated by qRT-PCR, with the expression of *DsRed* to examine differences in the integration marker for each transgenic line. Similar experiments were performed for our granulocyte promoter constructs, LRIM15 (**d**) and SCRASP1 (**e**), driving *GFP* expression under the control of the respective hemocyte promoter. Analysis of *CFP* expression was used to examine differences in the integration marker for each transgenic line. Data are displayed relative to *rpS7* expression with bars representing the mean ± SE of three or

more biological replicates (displayed by dots, N=>3). Statistical analysis in **a** was performed using a one-way ANOVA with a Tukey's multiple comparisons test, while the data in **b**-**e** were examined using two-tailed unpaired t tests. *P* values or adjusted *P* values are displayed in the figure where applicable. ns, not significant. Source data are provided as a Source Data file.

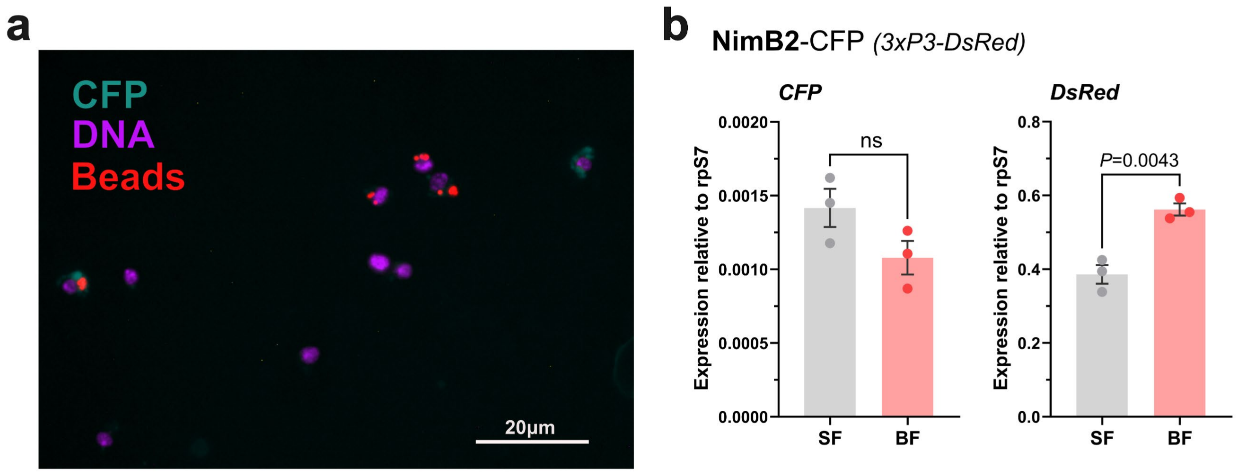

Supplementary Fig. 4. Additional characterization of the NimB2-CFP line. The *ex vivo* examination of NimB2-CFP expression in perfused hemocytes was below the limit of detection and unable to distinguish potentially labeled cell populations from background levels of fluorescence (**a**). The injection of fluorescent beads prior to perfusion allows for identification of phagocytic granulocytes. **(b)** The expression of NimB2-driven *CFP* or that of the *DsRed* integration marker were examined under sugar-fed (SF) or at 24hrs post-blood feeding (BF). Data represent the mean ± SE fold change expression of three independent biological replicates (N=3, dots) of pooled (n=~10) whole adult female mosquitoes, with significance determined using two-tailed unpaired t tests. *P* values are displayed in the figure where applicable. ns, not significant. Source data are provided as a Source Data file.

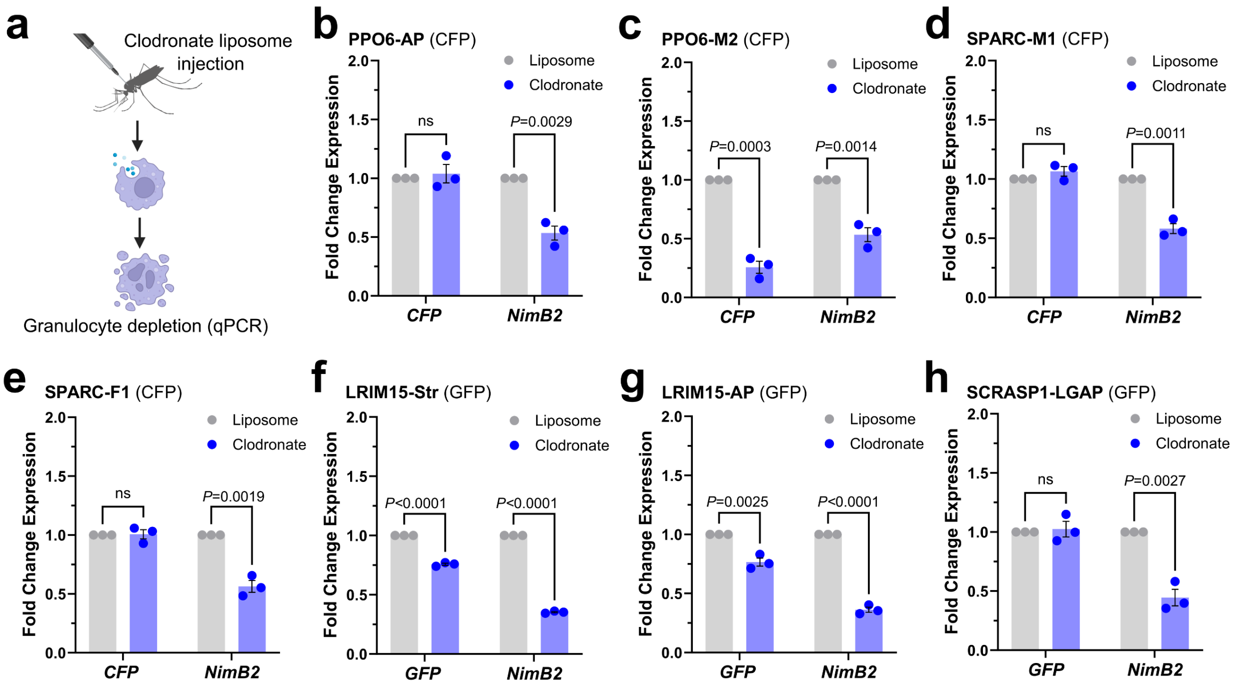

Supplementary Fig. 5. Gene marker expression in response to clodronate liposome injection. To determine the specificity of candidate hemocyte promoters in phagocytic granulocyte populations, we examined the expression of fluorescent markers driven by the respective candidate hemocyte promoters using qPCR as a proxy for granulocyte depletion (**a**). Whole mosquitoes from each PPO6 (**b**, **c**), SPARC (**d**, **e**), LRIM15 (**f**, **g**), and SCRASP1 (**h**) transgenic lines were examined 24hrs after injections with clodronate- or control liposomes by examining the expression of *CFP* or *GFP*. The expression of the hemocyte marker *NimB2* was used as a positive control. Data represent the mean ± SE fold change expression of three independent biological replicates (N=3, dots) of pooled (n=~10) whole adult female mosquitoes, with significance determined using multiple two-tailed unpaired t tests with a Holm-Sidak correction for multiple comparisons. Adjusted *P* values are displayed in the figure where applicable. ns, not significant. The experimental overview in **a** was created using BioRender: Smith, R. (2025) https://BioRender.com/vr4fplw. Source data are provided as a Source Data file.

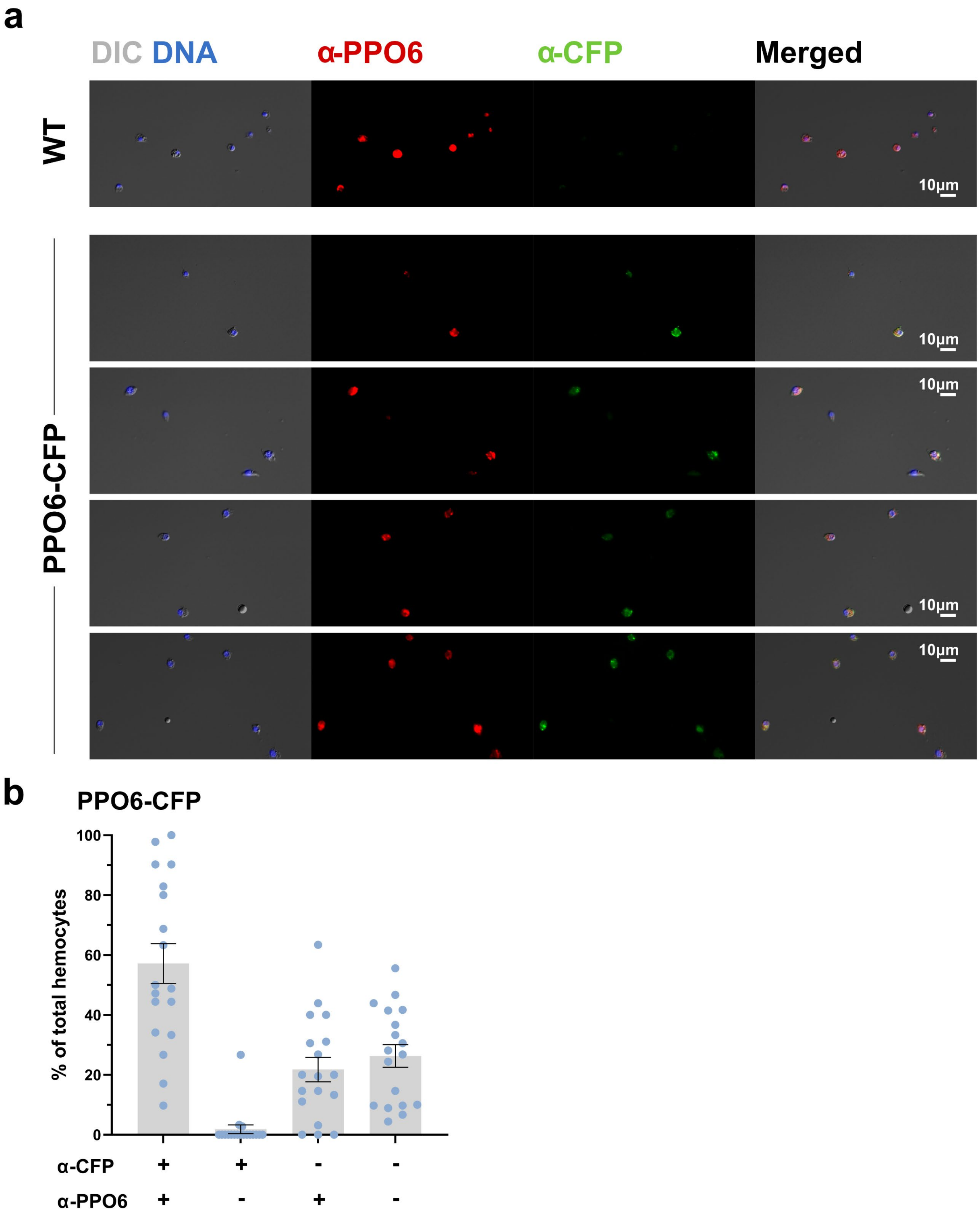

Supplementary Fig. 6. Co-localization of CFP and PPO6 in PPO6-CFP transgenic mosquitoes. Perfused hemocytes from wild-type (WT) and PPO6-CFP transgenic *An. gambiae* mosquitoes were examined by immunofluorescence using PPO6 (α-PPO6) and CFP (α-CFP) antibodies (**a**). Hemocytes from the wild-type strain display PPO6-positive

signals but lack CFP fluorescence. The percentage of fixed cells positive for one, both, or neither marker was then quantified in individual mosquitoes (n=18). Individual data points (dots) are summarized from three independent experiments as the mean ± SE. Scale bars represent 10μm. Source data are provided as a Source Data file.

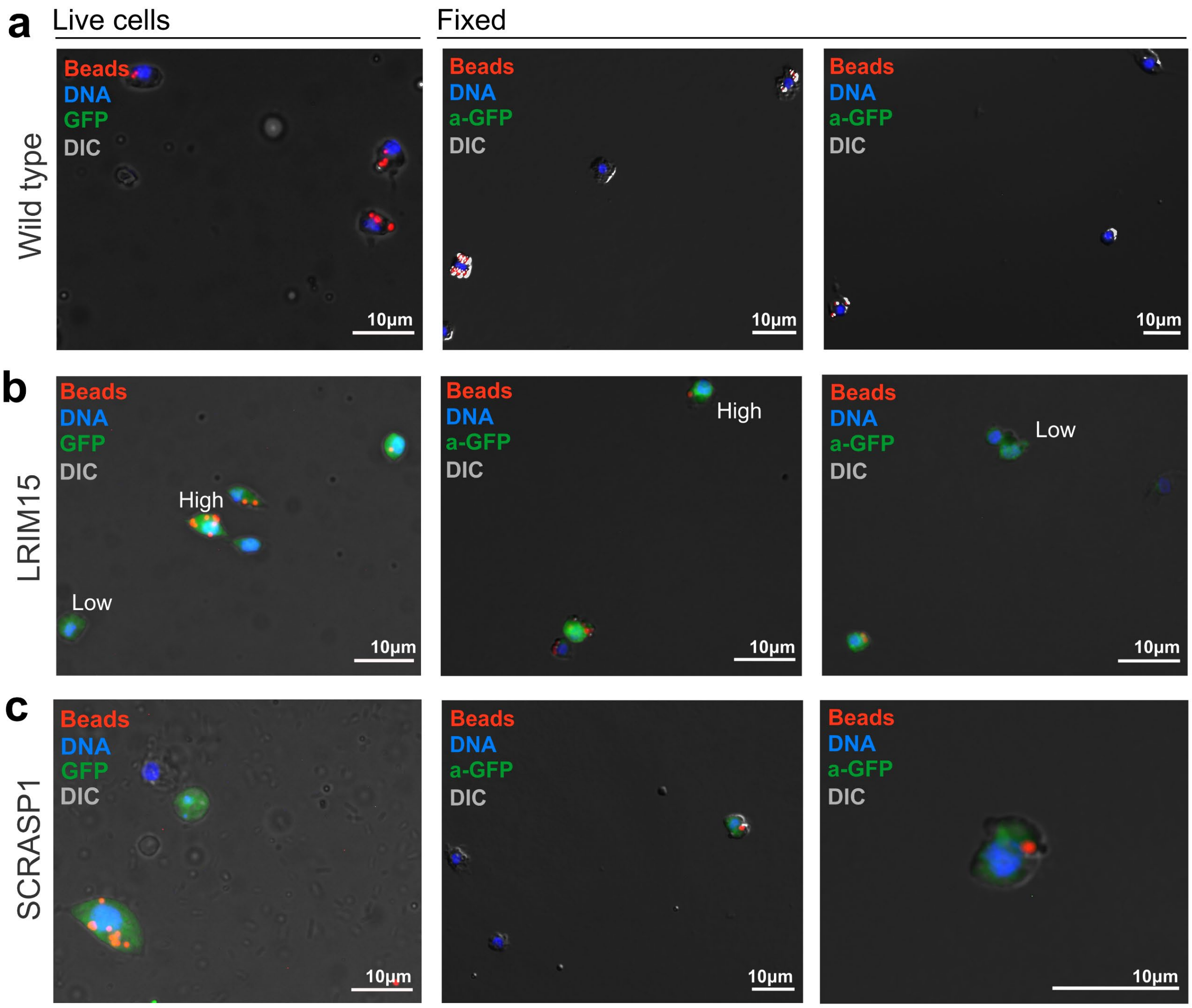

Supplementary Fig. 7. Visualization of LRIM15- and SCRASP1-GFP cells under native and fixed conditions. Perfused hemocytes from (**a**) wild type, (**b**) LRIM15, and (**c**) SCRASP1 mosquitoes were immediately observed under a fluorescent microscope as live cells or were fixed, then followed by immunostaining with a GFP antibody (a-GFP). Prior to perfusion, mosquitoes were injected with a solution containing 2% fluorescent beads (red) and 1 mM of Hoechst 33342 (blue) to define cell populations and identify phagocytic cell populations. Images were taken using the same exposure time (1000 msec) which was defined based on autofluorescence in the wild type (Keele) background. Scale bar: 10µm.

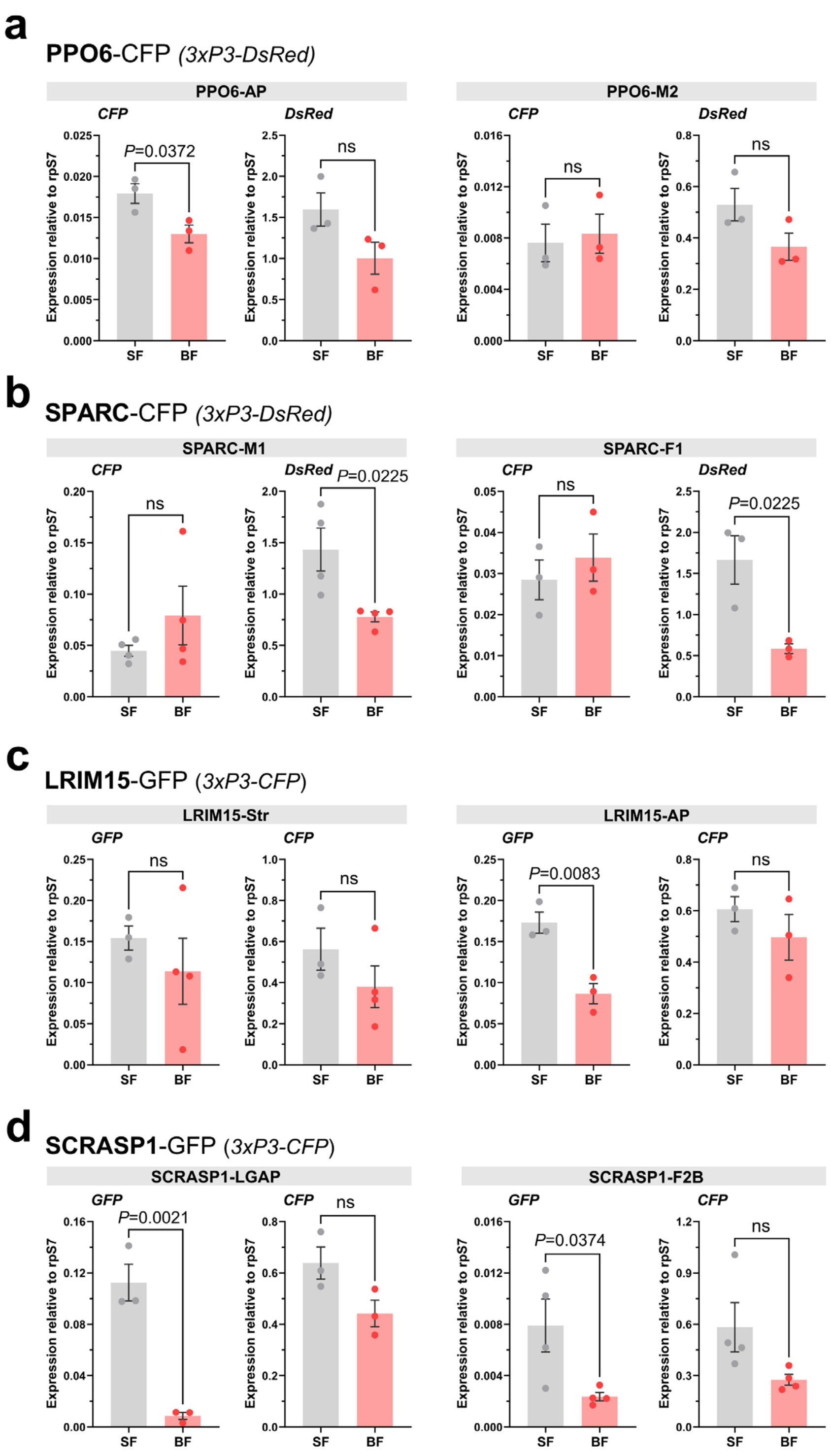

Supplementary Fig. 8. Hemocyte-specific marker gene expression responses to blood feeding. The expression of marker genes (*CFP*, **a-b**; or *GFP*, **c-d**) were examined for PPO6 (**a**), SPARC (**b**), LRIM15 (**c**), and SCRASP1 (**d**) transgenic lines under sugar-fed (SF) or at 24hrs post-blood feeding (BF). In addition, the expression of the integration marker (*DsRed,* **a-b**; or *CFP*, **c-d**) was used as an internal control. For each promoter

construct, individual transgenic lines are examined for comparison. Expression data are displayed relative to *rpS7* with bars representing the mean ± SE of three biological replicates (N=>3, dots) of pooled (n=~10) whole adult female mosquitoes. Data were analyzed for significance using two-tailed unpaired t tests. *P* values are displayed in the figure where applicable. ns, not significant. Source data are provided as a Source Data file.

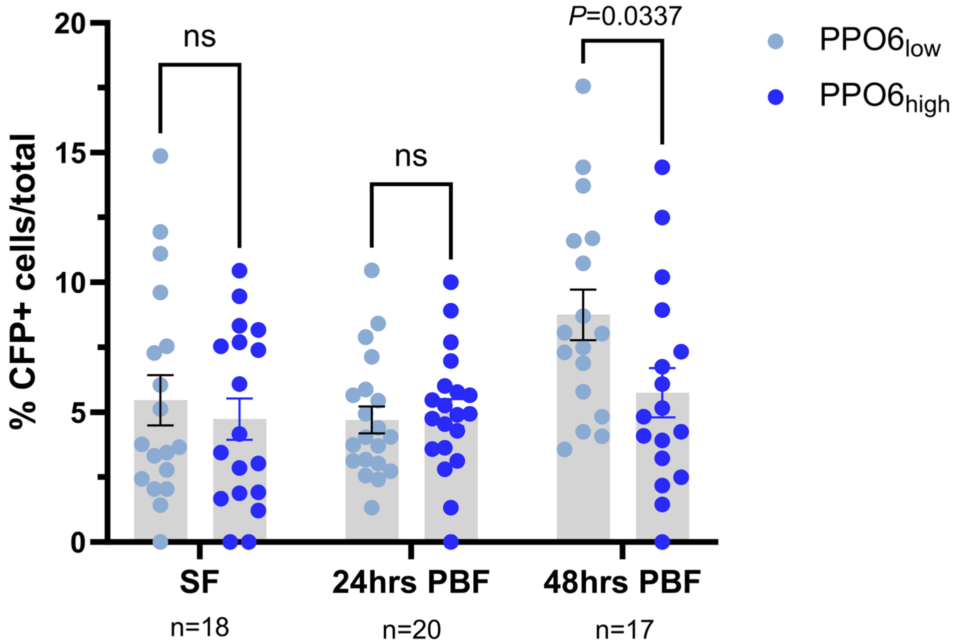

Supplementary Fig. 9. Blood-feeding promotes a shift in PPO6^{low} proportions. The percentage of PPO6-CFP cells with low (PPO6$^{low)}$ or high (PPO6^{high}) CFP fluorescence are displayed as the percentage of total hemocytes. Data from individual mosquitoes are represented by dots and represented as the mean ± SE of three independent biological replicates. Statistical significance was determined by a two-way ANOVA followed by Sidak's multiple comparison test. Adjusted *P* values are displayed in the figure where applicable. ns, not significant; n=number of individual mosquitoes examined. Source data are provided as a Source Data file.

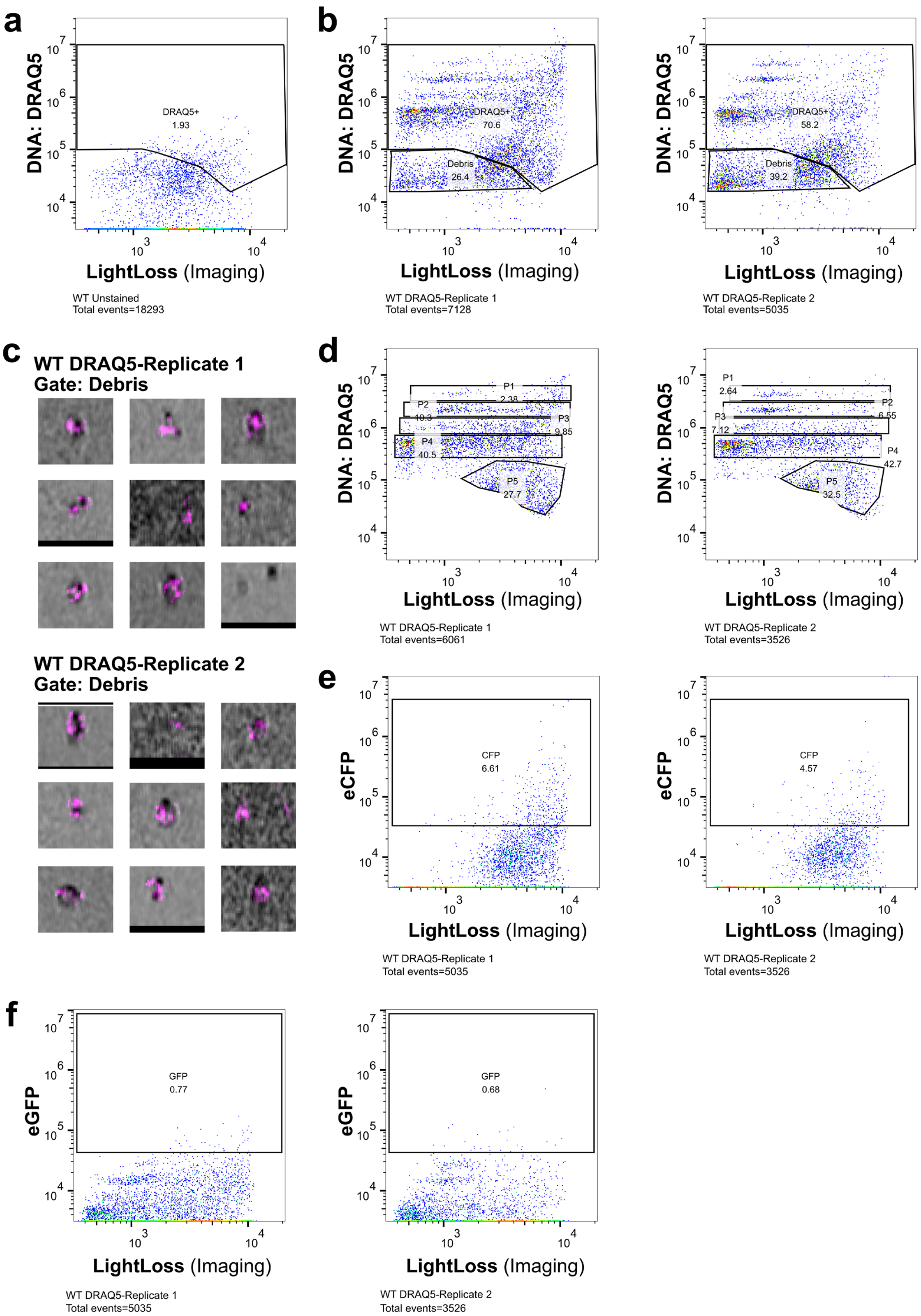
a
DNA: DRAQ5
DRAQ5+
1.93
LightLoss (Imaging)
WT Unstained
Total events=18293
b
DRAQ5+
70.6
Debris
26.4
WT DRAQ5-Replicate 1
Total events=7128
DRAQ5+
58.2
Debris
39.2
WT DRAQ5-Replicate 2
Total events=5035
c
WT DRAQ5-Replicate 1
Gate: Debris
WT DRAQ5-Replicate 2
Gate: Debris
d
P1
2.38
P2
10.3
P3
9.85
P4
40.5
P5
27.7
WT DRAQ5-Replicate 1
Total events=6061
P1
2.64
P2
6.55
P3
7.12
P4
42.7
P5
32.5
WT DRAQ5-Replicate 2
Total events=3526
e
eCFP
CFP
6.61
WT DRAQ5-Replicate 1
Total events=5035
CFP
4.57
WT DRAQ5-Replicate 2
Total events=3526
f
eGFP
GFP
0.77
WT DRAQ5-Replicate 1
Total events=5035
GFP
0.68
WT DRAQ5-Replicate 2
Total events=3526

Supplementary Fig. 10. Determination of gating for flow cytometry analysis. Prior to flow cytometry analysis, controls were performed to apply the proper gating of cells for further analysis. Unstained cells from wild-type *An. gambiae* were used to set threshold values (**a**) for gating for DRAQ5$^+$ positive events (cells) (**b**) to remove any autofluorescence background or cellular debris (**c**). Wild-type cells exhibited five distinct DRAQ5 signal patterns (**d**), corresponding to subpopulations P1-P5 that differ in their DNA content. Additional controls using wild-type DRAQ5$^+$ cells were used to set threshold values for gating CFP$^+$ (**e**) and GFP$^+$ cells (**f**). Of note, wild type cells contain a relatively high levels of CFP background fluorescence. For each experimental condition (**b**-**f**), data are displayed for two independent biological replicates. These gating strategies were used to define immune cell subpopulations in **Fig. 4** and in identifying transgenic immune cell populations in **Fig. 5**.

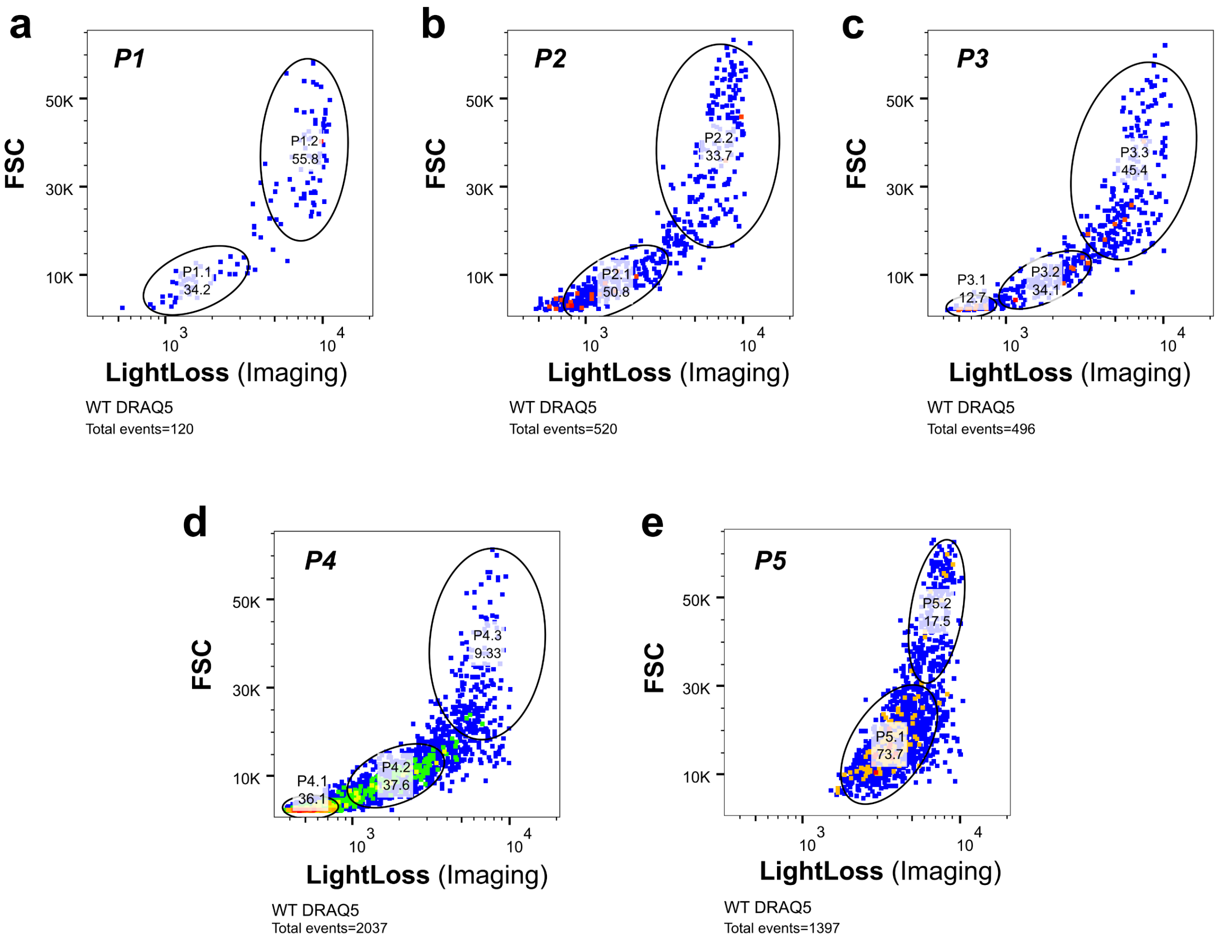

Supplementary Fig. 11. Classification of immune cell subtypes in the P1-P5 DRAQ5 clusters. Initial sorting of DRAQ5+ cells identified 5 cell clusters, P1 through P5, based on DNA content or ploidy (P). Further examination of the P1 (**a**), P2 (**b**), P3 (**c**), P4 (**d**), and P5 (**e**) clusters by size (FSC) and axial light loss reveals additional subpopulations for each cell cluster. Subpopulations are defined by circles, with the percentage of cells displayed for each subpopulation (of total). These gating strategies were used to define the immune cell subpopulations in **Figs. 4-6**.

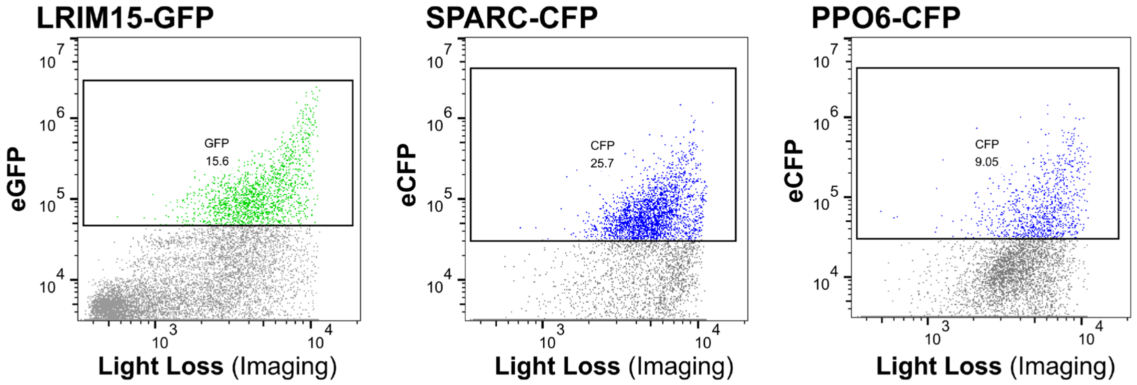

Supplementary Fig. 12. Identification of fluorescent hemocyte populations by flow cytometry. Representative scatter plots of fluorescent hemocyte distributions in LRIM15-GFP, SPARC-CFP, and PPO6-CFP transgenic mosquito lines. Gating strategies display cell events considered to be either GFP^+ or CFP^+ as presented in **Fig. 5**.

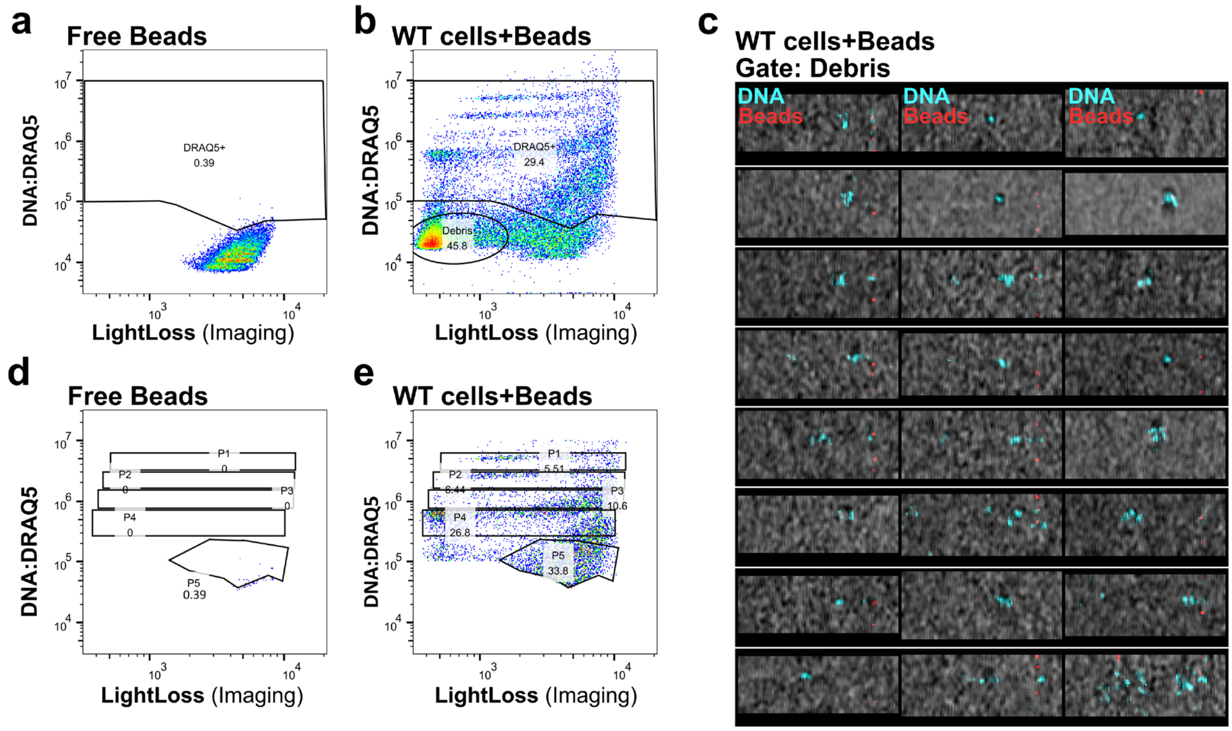

Supplementary Fig. 13. Determination of gating using fluorescent beads in flow cytometry. Prior to flow cytometry analysis, controls were performed to apply the proper gating of fluorescent beads for further analysis to examine phagocytosis. (**a**) A fluorescent bead-only sample was used to distinguish cutoffs for bead (red) and DRAQ5 (far-red) signals. (**b**) Mosquito perfusates were examined after the injection of beads to identify phagocytic cells. Cells were stained with DRAQ5, with events identified as immune cells (with or without beads), free beads, or cellular debris which was confirmed by imaging (**c**). Using this gating methodology, no bead signal was detected within the P1-P5 groups (**d**), while enabling the gating of immune cells (with or without beads) according to DRAQ5 signal (**e**). The displayed gating strategies are used to define differences in “free beads” with those of phagocytic cells displayed in **Fig. 6**.

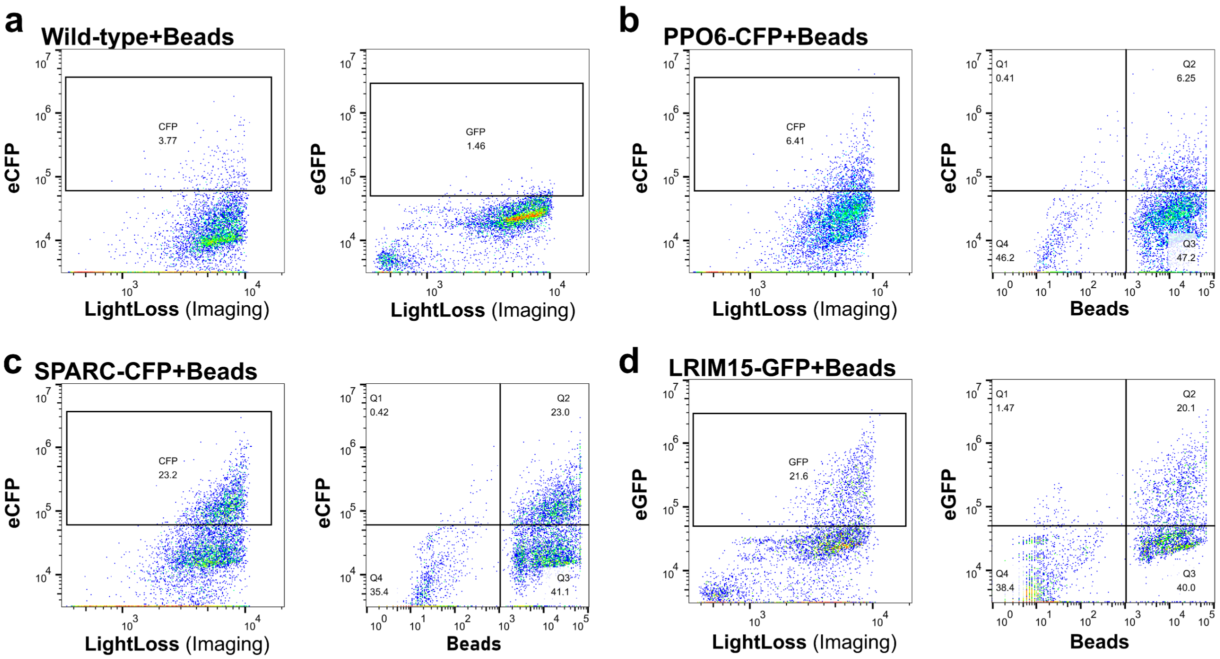

Supplementary Fig. 14. Gating thresholds for analysis of phagocytosis in transgenic lines. Prior to flow cytometry analysis, controls were performed to apply the proper gating of CFP and GFP fluorescence to perform phagocytosis assays in PPO6, SPARC, and LRIM15 transgenic lines. Gating thresholds were set for CFP and GFP fluorescence in wild-type mosquitoes injected with fluorescent beads (**a**). When these gating strategies are applied, phagocytosis was examined in PPO6-CFP (**b**), SPARC-CFP (**c**), and LRIM15-GFP (**d**) lines. The displayed gating strategies are used to define either CFP^+ or GFP^+ transgenic phagocytic immune cell populations that have taken up one or more beads via phagocytosis for the data presented in **Fig. 6**.

Supplementary Table 1. Sequences of candidate hemocyte promoter constructs.

Putative promoter sequences of genes-markers expressed in *Anopheles gambiae* hemocytes. Letters highlighted with gray indicate the annotated 5' Untranslated Region of each gene.

1. **>AgNimB2**

ACCTCCTCCACTACCACTTCAACAACCGAAGCTACTGGACAAATGCTACCAAACGCGAC
CTACGCCGAATCGTACCACGAGTACCTTCCCAACAACACCGAGATGAGCATGGTGCTA
ACAAAAGGTCACTCACGAACTGCCAGCAACGAAAGCATGTACGAGCTGGTGACGACTA
CGTCACGCGTCGAGATCACCTTCATCGATACGGCGGTGAAGAAAATTGAGGATCCCTC
CACGATCAACCATACAACGCCGGAGGAGGAGCCCGCTTCCCTGTCTGTAGAAAGCTCC
AACGAAACGGACACCCAACTGGTGTACTTGGAATCGGAGAACCATCCCGTTCTGGTGG
GCATAACCGAAGATGGGAGCGTGCAGAGTGAGGCAAATGATCGACAGGCGTTCGTCA
CGATCTCCTGCTTGCTGATCACACTCACCCTGCTAATGTCGGTGGTCATCTATATGAAG
CGTTTGAATCGCAAAACGCTTGCCAAACCTGCCGCTAGTGTGCCACCGCCGGTGAAGA
TTGTTGACGAGAGCGTCCAAACGGGGAATGATTCGAACCGCTCGTCGGATCCACTTCC
CGATCTGCCCCATGTCACCTACACGCGGGTAAAGCCAAAACTGCAACGAAACGGTGAT
ATCGGTAAGTGTATATAGAAGCAAGTTCTCCTACAATCTAACAGCAAGGCGAATCGTTA
AAACTAACTATTATCTTCCCAGAACACTATGATGTTCCGCCGAATAATAGTTTCATCCAT
CGTGCAAAGTCAGCATCCCCTTACAACTACAACTTCTCGGTCAACCAAAAGCAAGCTCC
CAGAAAGTATAGCCTTGAGCATATCTACGACGAAATTCAGTATCCCCCGTTGGCGGAAC
TGACGAGCAGTACAGCAACGCAGTCCGATAAGCCGACGTTGCAGCCGGGTGAGAAGG
AAGACAGTTATTCCAAACCGATGATTCTGGCGTAGGATAGGGCTAAGTTTGCTTATATG
TGGTTCTTATGTTGAACTAAATAAATGTATTTTTTTAACTTACCATAAAATAGGAATTGTT
GGTTACAGCGTGAGTATCGTCCATCTTCAATAATAGCTACCTCGTCTAGAGTTTAAATTA
TCTCAATTTTTTTTCTCTTGGCATTCGCTACGGCATGTATTTCTCACTCTTCCTTGTGGC
CGTTGCGCCACAGGTGACATGAAATATTAATTTCTATATCATCCATCAATCAATCGCTTC
ACAGCGCGCAACTTAATACATTCCATTCAGGATTCCCCTTCCATGCCTTTGCATTAAACA
TCTCGCATCACAATACATCAAACGTGAGTATGCCTGATGGGGCGCAGATGCCCGGTGG
CTTACTGTGTTGCGCCACTTCAAATCGCACCCGAGATCGGACTTTTAAGAATAAGCACA
TCACGTCATACGAGACGTTCGCCCACACACCACAACGTTTCGGTGCGATATCACATCAT
GCCAAAGAACGTCACCAGCCGTACCGCGACGAGTTCTCGCCATTAGGTTCTACTGACC
GTTTACTTTAATCCATCGCGAACCTTCCGTGGGGTAAGATTGAAGTTGAAGCTGTAGTG
ACCCCATTTCTTTACGATATTACACC

2. **>AgSPARC**

CCGCAATCACATCAGCTTCAAGAAGTACACGAGCCCCCCCTTCCTACATTAACGACCTT
AGCAGTACTTTTTGCGTATCAAACAAACACACACACACACTAACACATACAGATCGCCC
TTAATTACTTCACCGCATGTGCAAATGTCGGCAAGCGAATGAGGTTTGCAGGGACGGC
CGTCCATCCGGTCCATTCGAAACTTCAGAACACACTTCGGCACATTCTTCGGCTGAGG
AAACGAGCGCGGGAGCGGGCGCGCGCACGCAATCTCACATCTCATACACGAGCTA
ATCATCCGGCGGGGTCTTATGGTTTTGTCTTTAACAGTTCCCGCCCGCCCTTCCCCAGA
CCCCGCCGACTGTGGAGGGAGTGTGGCGAGTGTGTTCTCGCTTGCTCCGCTTGGCCG
GGAAGAGTCGTCGCATCACCGGGACGCCCACGAGATCCCATCCCGGCGGGTTCGGGT
CGACTCTCTGTGCCCGAGTCGAGTCAGTCTGGCTCTAACGATCGTGCCGATCGGACGC
CACCAAATCGGGTGCAAGAGACGTGCTTCCGGAGCTACCGCAGCTACCGTGTAGTGTT
ATTGTTTACTGGTGCACCAAGTCCTTGGGTCTTTGGGAACATTCTGTGCTTCAACGATA
TTCACCAACAAGAAACAGAAATCTTCAAAGTAAGTGTAGATTAAAGTTTGGAGTAGCTG

GGTGAGAGATGTTGGGAACTGTGACCTTACTCCTCAAGCCTGTTGGGAGTCGTTAGTC
CCCTGGAACCCTATTCAAAGTAAGTTCTGTGTACTGGGTTTGAATTGTTCCACGCTGTG
CCTAGCAATGGTTACATCTTCAATCGGACCTTATGTGGTCGCTTATCGCGGTCCGGTAC
GGATGCGACAAGTTCCTTCCAACGGTCCAAGCCTAACCTTCAATCAAACTGATAACCTC
GCGCGCTCTGAAACCGCGGCATCTTGCGGCGGAAGCAGTCTCCACACCGAAGCTGCC
AGTGTTACCGAATGTCGCCCCGAAACGGTACACAGGATGTTACTTCACACCACAGACC
AACGTCAGCAATTAATCGCTTGCGCAAACGTCTCCTCTGCCAGCGCCAACCAGACACC
CTCACGGGGGATTTTGAAACTGTAAACGCTCCCGGACGGTTGTGGCAGGACAATGTAC
CGAGGGGGGAGAGGGGGGGGGGAGTGTACGGATACACCGATGCTCAACTCGGTACA
GCACTGTTTACAGACAACTCGGGTGTCGTTATAAATAACCCACCGGTGGAGGTTTCGG
TTGGTTTTCGGTTTTTGGAAACCTCTCCATTGCGGGTCCGATACCGAGAATCAAAGGGT
GATGTCCGGGTAAGGGGACGGGATTCCGAAGGCAGGATCTCAAGTGCATCTGCCGAC
GAGGGGACGGGCTACGTTAAGCTATGTCTATGCTTCACTTTAACGACCACTCATTTCCC
TAAGTGCAGCGTGCGGTCGGCAACTCCAGAAACTCCATGCAAAGCCCTGCTAGGGATT
GTTTGGATAATATCGCTCATCAAGCTGGAGAGTTCGAGGGCGGAGATTCATCATCAAAA
CAGTACTTCTGGTACAGTATTATTACTGTTTGTCAGTGCAGTGGTATGTCCCCTTTCGGT
TCCAGGGGGTTTGTCCCTTTCAGCGAACTCTCAAACCCAAACAATCTCTAACGAAAGCA
TAACCGAAAGCAACCGCAACAAAACACAAACACTTGCCTCTCCGTTGTTGCCACGCGC
TTCTCCTAAACGCATCGTATGACATCAGCATTTGGCAGGACGGTTGTCGGTTTGATTTC
CTTGCCCATGTGGCCCGTTTTGCATCTTGCGCTTTCCTGCGCTCCGCACTTGCTACGTC
GCAACAAAATTCGCTCAGACTGTGTTCGCTGTGTGTGTGTGTTTTTTATCGCATCGGAA
GTACGCTGCGGTTGCGCCACTTCCGCCTAGAACGCCCTCCCCCACTCGTTCTGACACA
GATTTGTGGCACATGTGTGCCTTGGCTAGAGCGGCGACAACGTTGTAGCTTTAATAAG
CAGCTGGCTGGTGAGATTTTGCTTCCCACTTCCCACTCCATCGCTCCAATGATGATGAT
CTCAGAATGTTGCTTTTTTGTTTGCGGTGCCTTTCTTTCTTCTTGTTGCTCCAGTGCGCT
TCGTAACTGTCGCTCCAATAATATGCGAGCCTGGACGAATGAAGCGTGCGGGTCTTTG
CCGGTCCCGGCGCAGTGCACAAACTGCCAACATATGCACTGGCGAAACATTGTTTCTT
GCCCGTGGTCATGCGGAGCCGTGTCAGCGTTTTATTACCCGTCCGGGCAGAGATTGCA
TCATCGCGCAAGGAGGGTCTACCATCTCGGTCTACCATCGACCGACCGAGTGAGCAAG
TTTTACGATCTTAAACCAATCTTAATTACCGCGGCGAGCGCAACGTGTTAGGGCGGCTT
CCTGATACCGCCGTTTGCAATTCGTAGCAACGTAAAAGATGATGCACCATGGTCCTAAA
AACGCATCTGTTTCCTCGCACACACATGGAGCCTTGTGACGACTGGCACGTTTTAATGA
TGCAGAACGAGTGCTGAAAAGAAAGGTCGCCAACTGGAAGATAGGATTGAAAGGATTT
TTTTTTTAAAGAGCAAACTGTTACCTAAAGTGTGTCAGATACGGGTTAAAACGTGGAAC
CTTGGCAGATAATTGAACTCTTCAAACTTCCGCAAAGTCCTACAGGCACTCAGTGTCCC
ATCATTCGCATTCTTGAAGATCTTATTGTCGTACATCAATTAGGTATCTAAAACAAATAC
CTTTGGAGTCGCTCCCTAGACCTTAGATCTATCTCTTCTGACTCGTGCCTAAATAACTAC
GTTCTTATCTTATCCAAGTACTTAGACAGGAACGTTTGGACAGTTCCTTTAGCCTCTCCC
AACCCATACTCTACTCTCAGACTTTAACTTCAAATTCTCCACCAGGACCTAAAAACTGGC
CTATAAAGATCAATGTCTAGCAAGATTGTATCGCGAAATTGCGCAAATAATTTATTTTTG
TTGTTGCAAATAAATCCGTTGCATTTTATGAACTCTGGAAGAGATTATTATCGTCAAACG
ATATTAGCATCTCATGGTCGCTGTACTGATGGTAGTACTTGGTACAATTACTTGGCTTAA
GTTGTACTCTCTCTCTCTCTCTCTATCTCTCTCTCTCTCTCTCTCCTTCTCTCTCTCTT
TCTGTCTTTCTCTCTCTGTCTCTCTCTCTGTCTCTCTCTCCTTCTCTCTCTCTCTTTCTCT
CTCTCTCTTTCTCTCCCTCTGTCTCTCTCTCCTTCTCTCTTTCTCTTTCTCTCTCTGTCTC
TCTGTCTCTCTCTCTCTTTCTCTCTCTCGCTCTCTCTTTCTCTCTCCCTCTCTTTCCCCAT
ATCTCTCTCTTCCTCTCTCTCCTTCTCTCTTTTCCTCTCTTTTCTTCTCTTTCTTTCTCTCT

CGTTCTCTCTTTTTCATTCAACTTCTCTCTCTCTCTCTGCCGCTCTATCTCTAATTGTAAA
TGCAATCAATTGTAAATGTCAACGCTATTTCGGTCGAACCTTATCTTGGGGTTTAGCCAT
TGGTCCTTCGAACCGGATTTTCCACTGGCATTGGCTCTACTAACCATCTAACTTCACCT
TCTCTCACCTTTCTTTCCCCCTCGCCGGCGTCTTGCCCCAGCTTTTGTGAAACGGGCC
GACGAACGGCG

3. >AgPPO6

CCTCATCGCTGGGAAGAATAAAGGAAGCCATTCAGTCCGGTTTCGCAATGGCAGTATG
TATTGTTGTGATATAAAGAAAAATTTTACCTTTTGGCAAAACCAAGCTAATGTTTATTAC
AGGCGGACGGGACACGTGTTCCTCTGGATCCTAAGAAAGGCATCGATATTCTTGGCAA
TATTATGGAAAACTCGATCCTTTCGGTCAACGTACCGTACTACGGTAATTACCACTCGC
TTGGCCACGTTCTCATCGGCTTTATCCACGATCCGGACAACCTGTACCTCGAGGGACA
CGGTGTGATGGGTGACTTTACGACGGCAATGCGTGATCCAACGTTTTACCGTTTCCAT
GGCCACGTGGACGATGTGTTTGATATGCACAAGCAAAAGCTTTCGCCATACAAAGCGC
ACGAACTGTCCTTCCCAGGTGTATCCATCTCGGACGCAACGGTGCAGATTACGAGCGG
TAAGGCGGCCAGAAATAGATTGCTAACCTTCTGGCAACGGACGCAAGTTGATCTGGGA
ACGGGGCTAGATTTCGGACCGCAAGGTAACGTGTTGGCAACCTTCACCCACATCCAGC
ACGCACCGTTTGCGTACCAAATTATGGTACAAAACGAAACGGCGGAGCAAAAGAAGGG
AACTGTTCGCATTTTCCTCGCCCCGATCTACGATGCGAACGGAGAGCAACTGTTACTGA
GCCAGCAGCGTCGGTACATGCTGGAGATGGACAAATTTGTCGTCAAGTGTAAGTATAC
ATTAAGTTGAGCAATACTGTTATGGTGCTGCAATCGTATCGTGTGTTTCCTTCAAGTACA
TCCTGGCGATAATCGGATCATTCGACGATCGGACCAGTCAAGCGTAACCATACCGTAC
GAAAGGACCTTCCGGCGAGTTGACGCTTCCAACATGCCGGGCACGGAGAGCTTCCGC
TTCTGCAACTGTGGCTGGCCCGATCATATGCTGCTGCCCAAGGGACATCCCGATGGTC
AACCGTTCGATCTGTTTATCATGATTTCTGATTACAAGGACGATGCTGTAAGCACCGGA
TTCAATGAGTGAGTACATTACGGAATCAGTATTGATGAACTCGGTTTTAACTTTTGAGTT
TTTTTTTAAATATCTTATGCTCATGTAGGAATGAAAACTGTAACGATTCACATTCATACTG
TGGTCTACGCGATCAGCTGTATCCGGACCGTCGTGCGATGGGTTTTCCCTTTGACCGA
CAGCCGGTTGCCCAGGATCACTTGATGAAGGACTTTGTGGGCAGGTTCCCCAATATGA
GTCGTACCGTAGCGGAAGTTATGTTCACCAACACTATCATTTCACGCACGTAAATGGCA
TCACGATAACCGATCGATGAACGATACTTGTGGCTGACCCGTATGATCGTTTTTTTTTAT
CAACGAGGAACATTATATTAAAATGCAATGGAAAAATGAGCAATAAAATATATTCATAAT
CAAGCAAATATATACCGCATTTCATTGCATTCTTAAAGTTATTTCTATTTTTTGCAAGTAT
TATGATTTTTTTCAACAATGCGTGTTATTTTCCTCAACCACCGATATGCAATATGGATAA
GGCACACACATTCCCAGTTATCGTAATCGCTTACCAAATGATTGGAATACCGCAAACAT
CCACAAAAATAGAACGGATTGATATGTTTTATCAAAGCTTCATGCATGTGCATACGCGT
GGTTTACATTTGCTTCCCATGTACTGCAACAATTGCAGATTACAATTGCATTGTACCATA
TCATTATCGCAGATGCAGTGGTCGTAAACCGCAAAAATGGCGATTGGAATTGGAAGAAA
TTGCATACAACCAGGCACCGGCACGTCAAGTGCAAATCAGAATGATATATAAACTATAC
CAACTACTTCTGTAGCATCACAAACGTGACTGTAACAGTGACTGGTGGTTCTCGTTTCG
TTCTCGCGCGTCTTTAAACACCGAATCGTTCCTTTAAGTGGAAAAACTAACGGATAATC
AAGCG

4. >AgLRIM15

AATTGGGAGAAACAACAGAAGAGCGCCTTACGTTTGTGCCTTAAACTGCTTGTTGTGGT
TTGATACGAAAAAAGGAGAGGAAAATGAAAATGTGTGATAAAATGCGTTTTCCCATACG
CTTTTCCTTTCAGCAGTAGTAGCTTCTATGATCGTTGTGATAACATAAGAAAAAAGAGAC
GTATTTTAAGAGCCAATGTGGAACCAACTGCGATAAGCATACCTACGATCGCGTTGAAA

GAACAACAGTTCAAAAGGTGGTGTGATGAAGCTTCACGCATTGAAGGTTATCGTGTGTT
TAATTTGAAAGCGAAACATCTTTATTCTTCCTTTCAATTCCTTTCATTCACGGTATTGTCT
ATCTTGCAATTGATTTGGCTTAGGCAACCAAAAAACATAATCGATGATCGAACGCTTGAT
AAACCATATAAAGAAGAGTTTGGAGTGCAAAATGTCGAATTGATGCACATGAATGGATT
TGCATGAGCATAGATTGCAGCAAACTACACATAATTAATCTTAAGGCTGTATAGAAATAA
AACTTATTCACATAACACATAATTCACATAATTTTCTCTCAGATATTTGCAACTTGTAGTT
TGACTTAAGCTACACATTTCTAATCAATTTTACACAACAACTCAATTCATTGTAATCATTT
TATTTCAGACATTTGGTTAAAGCTTCATAATGTATACCAAAGAATCTTTTTCATGTAATTC
GTTAACATTATACGAAAATTACATTATACTATAAGAGTCATACACATGTGTTTGAGTTTTC
TAAAGGTGAGAAGTTTTTGGGGCAAGTAAAATGCATTGCCGGCATCGCATCATGTTTGC
TATACCAAAGCTTTGAATAACACAAAATTATTCGAAAACCGTCTGACATAAAAAAGAGAA
AGAAAGAAAACTCATAAATGCTCCAATAAGCATCCAATTAATAACGTGATGTGAAATGTA
AAAGAACCACCCATATGTTAAGGTGACCAACAATTTAGAACGGTCTAATCCGCGAATAC
ATGGCGAACCCGAGAAAGCTAAATTAAAGTTTCATATAGTCAGAATTTTGAAGATTACAA
GGATTTCCCACGAATTTGACACCAACACAGATCCATAAGACATGTAGGTGCACACCCAA
TACTTAGTATGCTATTCGAATTTTTGTTAATCAAAATCAAATCACATCCAACTGTAAAAAG
AGAACATTATCATTTAAAACTATGGACTTTTCACATTGGATGCACACTGAGCGTTCCTAC
CCAAGGTAATGATTGATAGCTCCAACGTTTATTGTGACCAATCTGTTCATGGACATCCA
AGATCGACTGACCTATCACATTGCCAACAGATCTAAGAAAAAATCGCAATTTTGCACATA
AATGAAAGATCCCAGCAGCGCACGGAGATATTACATGAGCTACGATTCGCGCAAGATC
CAATGATCATCAGTTTGCCTAGGCTCACTGACTCACACCCGCCAACCATACCGGCTATT
TTGCATGCTTCTTTCGTTTCACCAACGATCTCGAATAATGTCCCATGACGCTTTATCATA
CACACAGAGCAGTGGCGCTACTGCATATGATTCGTTGATAGTTTTTTTTTTTTTATTCTG
CATCTCCAAGCATTGCTCCTTATCACACGAAGATGCAAACTGAACGGCATTGCATCAGG
GTCTGAGTTAGTGGCTCACTGTAAAGTGATGTGGGGGGTGGGGGGGGCTTCATTTGTT
TATGGCACCTTTCGCTTTTTTGTGTCAGAGAGGCTTCATTTGTCTATGTCGCAGTATATT
GCAAACACTCATTGCGATCATCTTCCAATTACGTCACATGGAAATGTTTGGTTTGAAGA
GATCGCTATCAATGCAAAAACCTCCGTCTACAAACAGCTGACCATAAGGAACGGGCGG
CTGTGTAAGGCAGCAACTTTTCAAGGGCCAAGATGCTCTCGATGCAGGAAAAGTGTAG
ACAAAACTGATAAGAACGCCCTCATATGCGAGTCGGTTCGAGAGCAATCAAGAGCTAC
ACTTCCTGGGTTCGTCAGTTTGCTCCTAGCAGTGTGACACTAAACACTTCCCCGTGAGC
GCTGTCCATAGTGTTTCCCTTGAAAA

5. >AgSCRASP1

CCAGATGCACGGGGATTAAACAATTCAAACAATAACCAAGACTCATAAAATCTTTATTTC
CTATTCCTAATACTATAACGAGTCCGTTTATCCTAGCCTAGCTCTACCGCACGAATGTGT
TTGCCCTCTCCCTTCGCTCCTCTGGTCAGCGACCCGGGAACTCACGCACATCCTTTCA
CGCACACATTATACGACAGCCGTCGACTGCTATCGGTGAGTGGTGCGCCCAGGTGGA
CAGAACTGCGTAGTCAGGTTGGACCACAATAGTATTCGTAATAGTGATGGGAAAAATGA
AGATTTCGTCGGAATCGATTCCGGTTAGCTCCGAAGTTTTCTGGAATCGATTCCGGATA
GTAGGTCCGGAATCAGTTTCCGGAATCGGCTCCGGAATCGGCTCCGGAATCGGAATC
GTCTCCGGAATCGGAATCGGCTCCGGAATCGGAATCGGCTCTGGAATCGAATTTGGCT
CCGGAATCAGATTCAACTCCGGAATCGGAATCGGGCTTCGGAATCAAAATCGGCTCCG
ACATCGGAATTGACTCCCAAATCAGAATTGGCTTCGAAACGGAGTCGGTTTCGGCATCT
TCATAGGAATAGGCGTTTGGGATCAATGATGCTACTTGTTGATAGCGACAAAGAATCAA
AATTTACTTGTATATGAATTCAAATGGAGATTGTTATTTCGCTTATTTATTATTTTCGCCC
GATTGTTATTTCGCCCGGACTTATTTCGCTTCCCACACCTCAATTCAAAAACTAATTCAC

ATTCCGGAGCTAACTCCATTTCTGGAGTCAAGCCCTACTGATTCCGTTACCGGCGCAAA
TTGCGTTTCCCAGGTCTCATCGCTACTTGCCTCCCATCTTAACGATCTTGCGCACCAAC
CAGTCAATTGAGCATTAATTGAACCCATTCTAGAAGCGTGCCCGACGGTGCTACCCCTT
CTTCAATGAATCGGGGCATTTCACCGCATCATCGAATGTGTTTCACTGGAACCGCGTAG
TGAAGTACCCTTCAGCGGGGAAAGTGCGACCGATTTGTAGCCATTTAATTGATTCATTA
AATAGCGTCAATTTATTCGTCTACGCGGGCGCGCGCTCGCGCTATCATCTTGCGCCTC
TTTCTATTCCGTACGCACGATGGCCATGATTCATCCCATTTTGTTCGGGTTTGTTTTGGG
GGATTTACCACCGTGCGTGTGAGTGTGAAAGAAAAACATTGTCTCAACTGGGTAGTGAA
GAGTTCTTAACGACGACACAGAGCCGTCGTAAGTAATCAACGCAAAGATGCTGATAAG
AATGAAGAGAGAGAGCGAGAGACGCATCTAGCCGATGCGAGCGACGACGAACTGAGC
TGGAAATTAAACACGAAGCGCGTCCGACTGACACTAAAACTGCCCGATATTCACTGGCT
CTTTCATGCCGGTATCAACATATCGATAATAGGAGGGACGTGATTTTTTGCGAGAACGG
TGGTGTAATTTCAAACACCTACCAAATGGTGTGTGAACCTTTATGGATCGTTATTCAAAC
CCGCGACACTTGGTGAAGGTGTTACGCCCGCTCGTGTTCTCGCTCAGGTCATTGAATA
GCACCATGGTGTAATATTTAGAGCATCTTTGAGTAGTTGAGCATGAAATGAAAATGAAAT
AATGAATCTTAAAATGCATATGACATTGGTTATGGCTTTCCCGCACTAAATGACCACTTG
GCACGATTAGTGTTGATGCGTCCATTCACTACACGAGTGACTCTTCATTCAAGCCGAAC
CAATTCCGTTGGTTCTTCGCGCCTTCTTCCAATTCCTTCGCGATCGTCGCTTCCTGTTG
TTGTGAGCTCGCCCGAACCGAAGCGACTGACCTTTCGCTCTTCCCTTCCCGAGGGTGG
AAGCATCTCTCCTAATCGGCACCAACTGATGATCGCTAGCCCAAGTCTGAAACGAGGG
TCGCGGTCACACGACTGCTCATTGTACGCTCGTAACTGGACCAAGCAGGACGTCCAGT
ATCAGTGGCTCTTCGTTTAGTGATCTTGTTATTTTTTTTTTTTTGTGAGAGAATATTGTGC
CATCACAGCCGCACCAGCTCGTGTCTGTGGTTTTGTGTATCTCCAGACAAAGATTAACA
GTGACGGGATTAAAAGATAAGACACGAAGGAACCAGCTTCGAACGATCTTTAGTTGGG
AGATTGGCGCAAGCGAAGATTTGCGCGAAAGCG

Supplementary Table 2. Comparison of gene expression and tissue specificity of transgenic lines.

Transgenic construct	Line	Hemocyte/rpS7	Carcass/rpS7	Hemocyte enrichment
PPO6-CFP	M2	0.37	0.02	18.5
	AP+	0.37	0.01	37.0
SPARC-CFP	M1	0.6	0.11	5.5
	F1	0.23	0.03	7.7
LRIM15 -GFP	Str	0.37	0.13	2.9
	AP+	0.5	0.29	1.7

-Hemocyte/rpS7 and Carcass/rpS7 display relative expression of the CFP or GFP marker with rpS7

-Hemocyte enrichment is displayed as the fold change between expression in hemocyte and carcass

Supplementary Table 3. Percentage of hemocytes expressing transgenic markers under naïve conditions.

Transgenic construct	% of cells ± SE (HC)	% of cells ± SE (FC)
PPO6-CFP	10 ± 1.14	8.7 ± 0.6
SPARC-CFP	45.4 ± 2.6	27 ± 0.87
LRIM15-GFP	34.3 ± 3.3	15.2 ± 1.44

-Hemocytometer (HC); Flow Cytometry (FC)

Supplementary Table 4. Primers used for the amplification of the hemocyte promoter regulatory regions.

Promoter	Gene ID	Forward (5'-3')	Reverse (5'-3')
SPARC	AGAP000305	gtacggcgcgccGCAATCACATCAGCTTCAAGAAG	gtacggccggccactagtgcgatcgcCGTTCGTCGGCCCGTTTC
PPO6	AGAP004977	gtacggcgcgccTCATCGCTGGGAAGAATAAAGGAAG	gtacggccggccactagtgcgatcgcTTGATTATCCGTTAGTTTTTCCACTTAAAG
LRIM15	AGAP007045	gtacggcgcgccAATTGGGAGAAAACAACAGAAGAGC	gtacggccggccactagtgcgatcgcTTTTCAAGGGAAACACTATGGACAG
SCRASP1	AGAP005625	gtacggcgcgccAGATGCACGGGGATTAAAACAATTC	gtacggccggccactagtgcgatcgcTTTCGCGCAAATCTTCGCTTG

Small letters correspond to restriction sites attached to the 5' end of each primer following the GTAC spacer sequence to enable the digestion of PCR products.

Supplementary Table 5. Primers used in splinkerette PCR.

Primer	Primer sequence (5'-3')
Splink-GATC-Top (*BglII*)	GATCCCACTAGTGTCGACACCAGTCTCTAATTTTTTTTTTCAAAAAAA
Splink-CGG (*MspI*)	CGGCCACTAGTGTCGACACCAGTCTCTAATTTTTTTTTTCAAAAAAA
Splink-Bottom-Universal	CGAAGAGTAACCGTTGCTAGGAGAGACCGTGGCTGAATGAGACTGGTGTCGACACTAGTGG
piggyBac LE#1	CAGTGACACTTACCGCATTGACAAGC
piggyBac LE#2	GCGACTGAGATGTCCTAAATGCAC
piggyBac RE#1	CGATATACAGACCGATAAAACACATGCGTC
piggyBac RE#2	ACGCATGATTATCTTTAACGTACGTCAC

Supplementary Table 6. Primers used for gene expression analysis.

Gene	Gene ID	Forward (5'-3')	Reverse (5'-3')
CFP	AGAP000305	ATCAGCCACAACGTCTATATCACC	TGTGGCGGATCTTGAAGTTGG
GFP	AGAP004977	AACAGCCACAACGTCTATATCATG	TGTGGCGGATCTTGAAGTTCA
DsRed	AGAP007045	CGACATCCCCGACTACAAGAAG	GTAGATGAAGCAGCCGTCCTG
rpS7	AGAP010592	ACCACCATCGAACACAAAGTTGACACT	CTCCGATCTTTCACATTCCAGTAGCAC
NimB2	AGAP029054	CAATCTGCTCAAATGGCTGCTTCCACG	GCTGCAAACATTCGGTCCAGTGCATTC
