# Supplementary material for: Characterization of Anopheles gambiae immune cells through genetic and functional immunophenotyping
Source: Nat Commun. 2025 Dec 3;16:10875. doi: 10.1038/s41467-025-65895-6 (PMC12675505; doi:10.1038/s41467-025-65895-6)
Supplement: Supplementary file 2 — Reporting Summary [file 41467_2025_65895_MOESM2_ESM.pdf]

nature portfolio

Corresponding author(s): Smith, RC

Last updated by author(s): Sep 30, 2025

Reporting Summary

Nature Portfolio wishes to improve the reproducibility of the work that we publish. This form provides structure for consistency and transparency in reporting. For further information on Nature Portfolio policies, see our Editorial Policies and the Editorial Policy Checklist.

Statistics

For all statistical analyses, confirm that the following items are present in the figure legend, table legend, main text, or Methods section.

n/a	Confirmed	
☐	☒	The exact sample size (n) for each experimental group/condition, given as a discrete number and unit of measurement
☐	☒	A statement on whether measurements were taken from distinct samples or whether the same sample was measured repeatedly
☐	☒	The statistical test(s) used AND whether they are one- or two-sided<br>*Only common tests should be described solely by name; describe more complex techniques in the Methods section.*
☒	☐	A description of all covariates tested
☐	☒	A description of any assumptions or corrections, such as tests of normality and adjustment for multiple comparisons
☐	☒	A full description of the statistical parameters including central tendency (e.g. means) or other basic estimates (e.g. regression coefficient) AND variation (e.g. standard deviation) or associated estimates of uncertainty (e.g. confidence intervals)
☐	☒	For null hypothesis testing, the test statistic (e.g. F, t, r) with confidence intervals, effect sizes, degrees of freedom and P value noted<br>*Give P values as exact values whenever suitable.*
☒	☐	For Bayesian analysis, information on the choice of priors and Markov chain Monte Carlo settings
☒	☐	For hierarchical and complex designs, identification of the appropriate level for tests and full reporting of outcomes
☒	☐	Estimates of effect sizes (e.g. Cohen's d, Pearson's r), indicating how they were calculated

Our web collection on statistics for biologists contains articles on many of the points above.

Software and code

Policy information about availability of computer code

Data collection	n/a
Data analysis	Graphical data visualization was performed using GraphPad Prism (version 10.4.2). Microscopy images were initially captured and processed using ZEN and Zen lite software (Zeiss) prior to final processing with Adobe Photoshop (version 26.7). Flow cytometry analysis was performed with FlowJo software (version 10.10.0). Figures were created using Inkscape (version 1.0.2-2) and in some instances were supplemented using images developed with BioRender (BioRender 2024).

For manuscripts utilizing custom algorithms or software that are central to the research but not yet described in published literature, software must be made available to editors and reviewers. We strongly encourage code deposition in a community repository (e.g. GitHub). See the Nature Portfolio guidelines for submitting code & software for further information.

Data

Policy information about availability of data

All manuscripts must include a data availability statement. This statement should provide the following information, where applicable:

- Accession codes, unique identifiers, or web links for publicly available datasets
- A description of any restrictions on data availability
- For clinical datasets or third party data, please ensure that the statement adheres to our policy

All raw data behind the displayed figures and analysis have been included as source files. In addition, data files for flow cytometry analysis are provided using Iowa State University's open data repository, DataShare (DOI: 10.25380/iastate.30192754).

Research involving human participants, their data, or biological material

Policy information about studies with human participants or human data. See also policy information about sex, gender (identity/presentation), and sexual orientation and race, ethnicity and racism.

Reporting on sex and gender	n/a
Reporting on race, ethnicity, or other socially relevant groupings	n/a
Population characteristics	n/a
Recruitment	n/a
Ethics oversight	n/a

Note that full information on the approval of the study protocol must also be provided in the manuscript.

Field-specific reporting

Please select the one below that is the best fit for your research. If you are not sure, read the appropriate sections before making your selection.

☒ Life sciences ☐ Behavioural & social sciences ☐ Ecological, evolutionary & environmental sciences

For a reference copy of the document with all sections, see nature.com/documents/nr-reporting-summary-flat.pdf

Life sciences study design

All studies must disclose on these points even when the disclosure is negative.

Sample size	For all mosquito experiments, sample sizes (defined as the number of individual mosquitoes examined) are depicted for each experiment and were determined based on previously published studies in which similar methods were employed.<br><br>For gene expression studies, pooled mosquito samples consisting of ~10 larvae or adult female mosquitoes were used per samples for each experimental replicate. Hemocyte gene expression was examined by collecting the hemolymph perfusions from >30 individual adult female mosquitoes. Microscopy experiments were performed on ~15-20 individual mosquitoes. Flow cytometry analysis was performed on ~40 mosquitoes per sample.
Data exclusions	No data were excluded from our analysis. Due to the lack of expression in our Nimrod B2 lines, or the minimal expression of SCRASP1, these transgenic lines were not included in our flow cytometry analsyis.
Replication	Gene expression and flow cytometry experiments were performed in three or more independent experiments.<br><br>Microscopy analysis of individual mosquitoes was performed in two or more experiments.<br><br>All attempts at replication were successful.
Randomization	Mosquitoes derived from the same batch/cohort were pooled and then used for experiments/treatments for each biologically independent sample. For each experiment, larval or adult mosquito samples were chosen at random for downstream manipulations, treatments, or analysis.
Blinding	For in vivo mosquito experiments, blinding was not possible due to limitations in the number of qualified personnel able to perform experiments.

Reporting for specific materials, systems and methods

We require information from authors about some types of materials, experimental systems and methods used in many studies. Here, indicate whether each material, system or method listed is relevant to your study. If you are not sure if a list item applies to your research, read the appropriate section before selecting a response.

Materials & experimental systems

n/a	Involved in the study	
☐	☒	Antibodies
☒	☐	Eukaryotic cell lines
☒	☐	Palaeontology and archaeology
☐	☒	Animals and other organisms
☒	☐	Clinical data
☒	☐	Dual use research of concern
☒	☐	Plants

Methods

n/a	Involved in the study	
☒	☐	ChIP-seq
☐	☒	Flow cytometry
☒	☐	MRI-based neuroimaging

Antibodies

Antibodies used	anti-GFP (DHSB-GFP-12A6], anti-CFP (1 mg/mL, Biosensis), anti-PPO6, goat anti-mouse Alexa Fluor 488, goat anti-rabbit Alexa Fluor 568
Validation	The GFP antibody was provided by the Developmental Studies Hybridoma Bank (https://dshb.biology.uiowa.edu/DSHB-GFP-12A6). This resource has been cited 31 times. Additional experimental validation in our lab confirmed signal in only GFP+ cells. Immunostaining was performed using a 1:50 dilution.<br><br>The CFP antibody was purchased from Biosensis (https://www.biosensis.com/anti-cyan-fluorescent-protein-mouse-monoclonal-919.html). Additional experimental validation in our lab confirmed signal in transgenic mosquitoes compared to WT mosquitoes. Immunostaining was performed using a 1:250 dilution.<br><br>An An. gambiae PPO6 antibody was previously developed by Muller et al (PMID:10206988) and was previously shared by George Christophides. Immunostaining was performed using a 1:500 dilution.<br><br>Secondary goat anti-mouse Alexa Fluor 488 (Thermo Fisher Scientific, #A-11001) or goat anti-rabbit Alexa Fluor 568 (Thermo Fisher Scientific, #A-11011) antibodies were used for immunostaining using a 1:500 dilution.

Animals and other research organisms

Policy information about studies involving animals; ARRIVE guidelines recommended for reporting animal research, and Sex and Gender in Research

Laboratory animals	All work was performed using larvae or adult female mosquitoes using a lab colony of Anopheles gambiae, Keele strain. As referenced in the methods section, this colony has been previously characterized (PMID: 28033418) and has been continuously maintained at Iowa State University since 2015.
Wild animals	n/a
Reporting on sex	Experiments were performed on female mosquitoes due to their implications in mosquito-borne disease transmission.
Field-collected samples	n/a
Ethics oversight	Experiments were performed under the approval of Iowa State University's Institutional Biosafety Committee (IBC) protocols #24-085 and #24-086.

Note that full information on the approval of the study protocol must also be provided in the manuscript.

Plants

Seed stocks	n/a
Novel plant genotypes	n/a
Authentication	n/a

Flow Cytometry

Plots

Confirm that:

☒ The axis labels state the marker and fluorochrome used (e.g. CD4-FITC).

☒ The axis scales are clearly visible. Include numbers along axes only for bottom left plot of group (a 'group' is an analysis of identical markers).

☒ All plots are contour plots with outliers or pseudocolor plots.

☒ A numerical value for number of cells or percentage (with statistics) is provided.

Methodology

Sample preparation	Mosquito immune cells (hemocytes) were perfused from adult mosquitoes and collected on ice. Samples for flow cytometry were prepared from a total of ~40 mosquitoes. Immediately after collection, samples were centrifuged at low speed, then the supernatant replaced by 1ml of 1xPBS containing DRAQ5 (1:1000). After incubation for 1 hour, samples were centrifuged then washed in 1ml of 1xPBS prior to sample analysis.
Instrument	BD Biosciences FACSDiscover S8 with CellView Imaging Technology
Software	Flow cytometry data were analyzed using FlowJo software v10.10.0
Cell population abundance	All raw data regarding the abundance of specific cell types (of total) is reported in supplemental materials. Data were examined from triplicate experiments. Data files for flow cytometry experiments are provided using Iowa State University's open data repository, DataShare (DOI: 10.25380/iastate.30192754).
Gating strategy	Cells were initially gated based on DNA content (DRAQ5), using image data to confirm the gating of individual cells. Cell gatings were based on cell properties such as size, shape, granularity, or fluorescence. Details of gating strategies is displayed in detail in our supplemental materials.

☒ Tick this box to confirm that a figure exemplifying the gating strategy is provided in the Supplementary Information.
